# The Manchester Chloroform Cases

**Published:** 1891-07-11

**Authors:** H. Woolley


### THE MANCHESTER CHLOROFORM CASES.

DEAR SIR,—In your article on chloroform in the last issue of THE HOSPITAL, there is one statement which I wish to correct—namely, that "the chloroform supplied by my firm is extensively used in America." I regret not to have clearly explained my meaning. What I had wished to convey was that the chloroform in question was made by the same process as that so extensively used in America.—I am, yours faithfully,

H. WOOLLEY.

